# Cytotoxic T Lymphocytes Regenerated from iPS Cells Have Therapeutic Efficacy in a Patient-Derived Xenograft Solid Tumor Model

**DOI:** 10.1016/j.isci.2020.100998

**Published:** 2020-04-06

**Authors:** Soki Kashima, Takuya Maeda, Kyoko Masuda, Seiji Nagano, Takamitsu Inoue, Masashi Takeda, Yuka Kono, Takashi Kobayashi, Shigeyoshi Saito, Takahiro Higuchi, Hiroshi Ichise, Yuka Kobayashi, Keiko Iwaisako, Koji Terada, Yasutoshi Agata, Kazuyuki Numakura, Mitsuru Saito, Shintaro Narita, Masaki Yasukawa, Osamu Ogawa, Tomonori Habuchi, Hiroshi Kawamoto

**Affiliations:** 1 Laboratory of Immunology, Institute for Frontier Life and Medical Sciences, Kyoto University, 53 Kawahara-cho, Shogoin, Sakyo-ku, Kyoto 606-8507, Japan; 2 Department of Urology, Akita University Graduate School of Medicine, Akita, Japan; 3 Department of Urology, Kyoto University Graduate School of Medicine, Kyoto, Japan; 4 Department of Medical Physics and Engineering, Division of Health Sciences, Osaka University, Osaka, Japan; 5 Dentistry and Pharmaceutical Sciences, Okayama University Graduate School of Medicine, Osaka, Japan; 6 Department of Medical Life Systems, Faculty of Life and Medical Sciences, Doshisha University, Kyoto, Japan; 7 Department of Biochemistry and Molecular Biology, Shiga University of Medical School, Shiga, Japan; 8 Department of Hematology, Clinical Immunology and Infectious Diseases, Graduate School of Medicine, Ehime University, Matsuyama, Ehime, Japan

**Keywords:** Cellular Therapy, Immunological Methods, Cancer

## Abstract

Current adoptive T cell therapies conducted in an autologous setting are costly, time consuming, and depend on the quality of the patient's T cells. To address these issues, we developed a strategy in which cytotoxic T lymphocytes (CTLs) are regenerated from iPSCs that were originally derived from T cells and succeeded in regenerating CTLs specific for the WT1 antigen, which exhibited therapeutic efficacy in a xenograft model of leukemia. In this study, we extended our strategy to solid tumors. The regenerated WT1-specific CTLs had a strong therapeutic effect in orthotopic xenograft model using a renal cell carcinoma (RCC) cell line. To make our method more generally applicable, we developed an allogeneic approach by transducing HLA-haplotype homozygous iPSCs with WT1-specific *TCR α/β* genes that had been tested clinically. The regenerated CTLs antigen-specifically suppressed tumor growth in a patient-derived xenograft model of RCC, demonstrating the feasibility of our strategy against solid tumors.

## Highlights

• Patient-derived xenograft of renal cell carcinoma was used in a cell-therapy model
• Cytotoxic T lymphocytes (CTLs) that target WT1-antigen were used as effector cells
• CTLs produced from iPSCs transduced with WT1-TCR genes showed efficacy in the model
• The present results demonstrate the feasibility of our strategy against solid tumors

## Graphical Abstract

**Figure:**
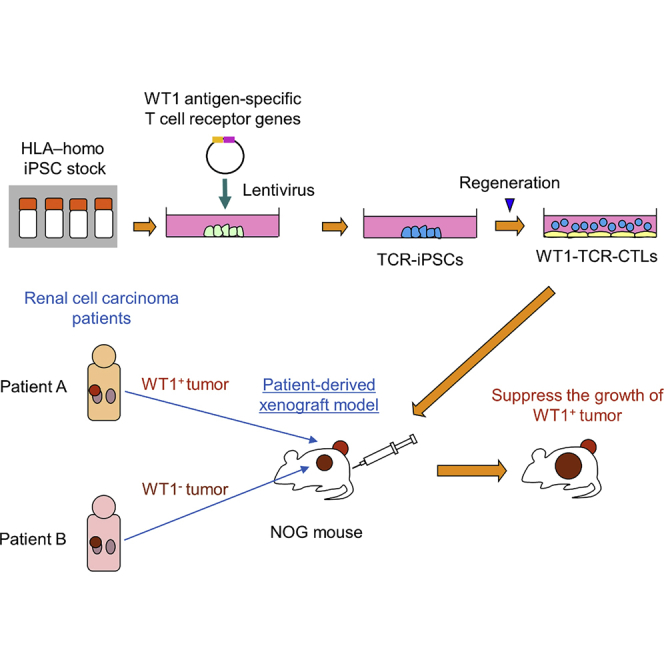

## Introduction

Recent remarkable advances in cancer immunotherapy have taught us that cytotoxic T lymphocytes can kill tumor cells. Immune checkpoint blockade therapies, such as with anti-CTLA-4 or anti-PD1 monoclonal antibodies (mAbs), have been shown to be effective against various types of cancer by enhancing endogenous anti-cancer immunity, and ultimately, cytotoxic T lymphocytes (CTLs) are thought to function as effector cells to kill cancer cells (Pardoll, 2012, Gong et al., 2018). Some strategies in adoptive T cell therapy, where T cells are collected from a patient and then given back to the patient after *ex vivo* activation, expansion or genetic manipulation, have also shown therapeutic effects against cancer (Rosenberg and Restifo, 2015). For example, Rosenberg and colleagues have demonstrated that transfusion of *ex-vivo* expanded tumor-infiltrating lymphocytes (TILs) was effective for patients with melanoma (Dudley et al., 2002, Rosenberg et al., 2011, Radvanyi et al., 2012, Besser et al., 2013). T cells that are genetically modified to express exogenous antigen receptors by gene transfer have also been shown to be effective (Morgan et al., 2006, Porter et al., 2011). One of such applications, in which peripheral T cells are transduced with a chimeric antigen receptor (CAR) gene that targets CD19, has shown dramatic efficacy against B cell leukemia/lymphoma (June and Sadelain, 2018). Transfer of *T cell receptor* (*TCR) α/β* genes targeting NY-ESO-1 or MART1 has also been shown to be effective against various tumors (Klebanoff et al., 2016).

These strategies of adoptive T cell therapy have mainly been conducted in an autologous setting. However, such an autologous approach is costly and time consuming and depends on the quality of the patient's T cells, sometimes failing to produce effector cells. To resolve these issues, it would be advantageous to develop a strategy conducted in an allogeneic setting, in other words, to prepare “off-the-shelf” therapeutic T cells (O'Reilly et al., 2016, Qasim et al., 2017). To this aim, we previously devised a method in which CTLs are cloned and expanded by using induced pluripotent stem cell (iPSC) technology. When iPSCs are produced from antigen-specific T cells (T-iPSCs), the rearranged *TCR α/β* genes are inherited by such T-iPSCs, and thus CTLs regenerated from the iPSCs should exhibit the same antigen specificity as the original CTLs. As proof of concept, we previously succeeded in producing iPSCs from human CTLs specific for the melanoma antigen MART1 and then in regenerating CTLs from the MART1-T-iPSCs (Vizcardo et al., 2013). We then improved our culture procedures and succeeded in inducing very potent CD8αβ type CTLs, and using this improved method, we regenerated WT1 antigen-specific CTLs (Maeda et al., 2016). These regenerated CTLs were able to prolong the survival of mice in a xenograft leukemia model where WT1-expressing human leukemia cells were inoculated into immunodeficient mice followed by transfusion of the WT1-specific regenerated CTLs (WT1-CTLs) (Maeda et al., 2016).

As the next step, we wished to apply our approach to a solid tumor. We decided to focus on renal cell carcinoma (RCC). RCC is considered to be one of the most immunogenic tumors, along with malignant melanoma and non-small cell lung cancer. In this context, even classical immunotherapies such as systemic administration of IL-2 or IFNα have shown therapeutic efficacy against RCC (Medical Research Council Renal Cancer Collaborators, 1999, Pyrhonen et al., 1999). Recent approval of anit-CTLA-4 and anti-PD1 mAb against RCC also supports the idea that RCC is immunogenic (Motzer et al., 2015, Motzer et al., 2018).

In the present study, we applied our method to RCC. We first demonstrated that regenerated WT1-specific CTLs cloned by our group expressing an endogenous WT1-specific TCR exhibited therapeutic efficacy against an RCC cell line inoculated into the kidney of immunodeficient mice. As a next step, we took advantage of methods that are clinically applicable in an allogeneic transfer setting: we first regenerated CTLs from HLA haplotype-homozygous iPSCs and transduced them with WT1-specific *TCR α/β* genes that had already been tested clinically (Tawara et al., 2017). Transfusion of these CTLs significantly suppressed growth of RCC in a patient-derived xenograft model, providing rationale for the clinical application of our strategy to treat solid tumors.

## Results

### Regenerated WT1-CTLs Exhibit Cytotoxic Activity against RCC Cells Expressing Endogenous WT1 Antigen *In Vitro*

**Figure 1:**
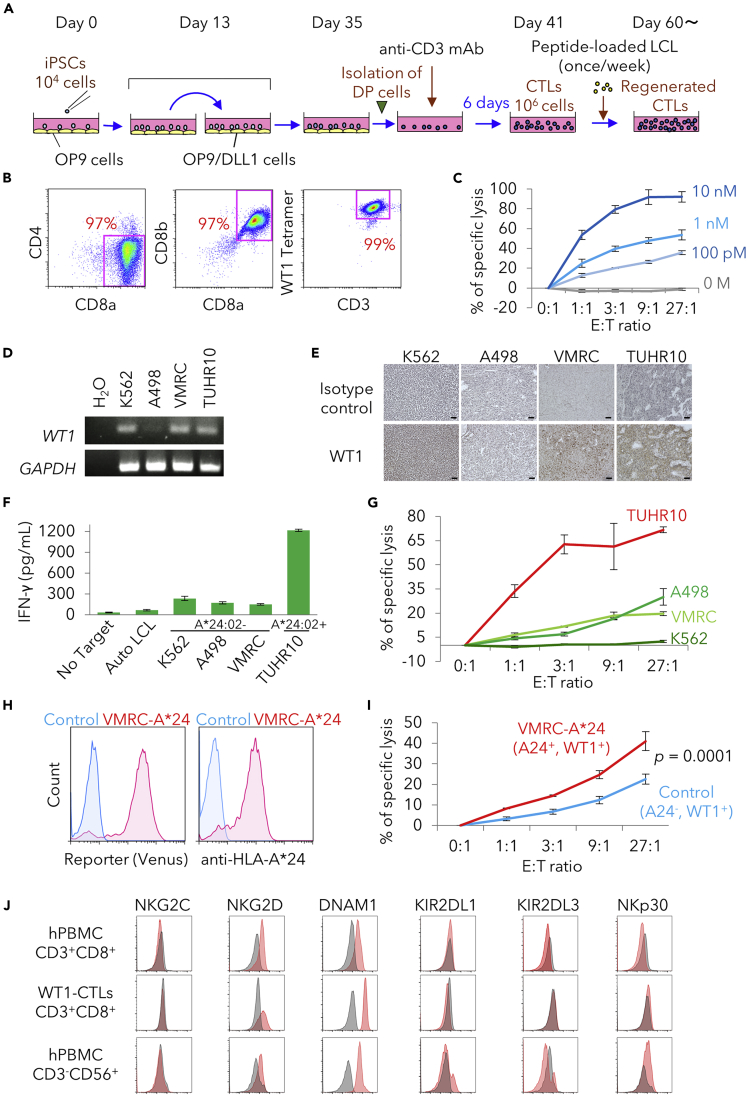
Regenerated WT1-CTLs Exhibit Cytotoxic Activity against RCC Cells Expressing Endogenous WT1 Antigen *In Vitro* (A) Illustration of the methods used to regenerate CTLs from T cell-derived iPSCs (T-iPSCs). (B) Flow cytometric profiles of WT1-specific CD8αβ T cells regenerated from clone #3-3 T-iPSCs. Results of one experiment are shown as representative of three independent experiments. (C) *In vitro*^51^Cr-release cytotoxicity assay of regenerated WT1-CTLs against peptide-loaded autologous LCL at different peptide concentrations. Results are presented as mean ± SD from biological triplicate experiments. (D) RT-PCR showing expression of the *WT1* and *GAPDH* genes in RCC cell lines. K562 was used as a positive control for WT1 expression. H_2_O was used as a negative control. (E) Immunohistochemical analysis for the expression of WT1 protein in cell lines. Scale bar, 50 μm. (F) IFN-γ production by WT1-CTLs in response to autologous LCL, K562, and three RCC cell lines. The effector-to-target (E:T) ratio was fixed at 1:1. Results are presented as mean ± SD from biological triplicate experiments. (G) *In vitro*^51^Cr-release cytotoxicity assay of WT1-CTLs against RCC cell lines at different E:T ratios. HLA-negative K562 cells were used as a control for NK-like cytotoxicity. Results are presented as mean ± SD from biological triplicate experiments. (H) Flow cytometric profiles of expression with HLA-A∗24:02 and Venus as reporter gene between VMRC cells and VMRC-A∗24 cells. (I) *In vitro*^51^Cr-release cytotoxicity assay of WT1-CTLs against VMRC cells and VMRC-A∗24 cells at different E:T ratios (∗p < 0.05). Results are presented as mean ± SD from biological triplicate experiments. (J) Flow cytometric profiles of expression with NK-related surface markers in peripheral CD8T cells of healthy donor, WT1-CTLs, and peripheral NK cells of healthy donor.

As a cell source to produce effector CTLs, we first used WT1-specific T-iPSCs (WT1-T-iPSC, clone name #3-3) that were originally established by reprogramming WT1-specific CTLs expanded from peripheral blood T cells of a healthy volunteer, characterized well regarding profiles as pluripotent stem cells (Maeda et al., 2016). CTLs were regenerated from #3-3 WT1-T-iPSC as previously described. Briefly, WT1-T-iPSC were co-cultured with OP9 cells and then transferred to OP9/DLL1 cells on day 13. CD4/8 double-positive (DP) cells generated on day 35 were isolated and stimulated with anti-CD3 mAbs for 6 days, followed by expansion using a WT1 peptide-loaded autologous B lymphoblastoid cell line (LCL) (Figure 1A). The regenerated CTLs were almost exclusively CD4^−^CD8^+^, CD8αβ-heterodimer, and WT1-tetramer^+^ (hereafter referred to as WT1-CTLs) (Figure 1B). The WT1-CTLs efficiently killed peptide-loaded LCL (Figure 1C), confirming our published results (Maeda et al., 2016).

To examine whether the WT1-CTLs are effective against solid tumors, we selected RCC as a target tumor and used three RCC cell lines, A498, VMRC, and TUHR10. TUHR10 cells express HLA-A∗24:02, to which the #3-3 WT1-specific TCR is restricted, but the other two lines do not. The K562 erythroleukemia cell line, which is known to express WT1 antigen, was used as a positive control for expression of the WT1 antigen and also as target cells for NK cell-like cytotoxic activity, because K562 is known to be sensitive to NK cell-mediated cytotoxicity due to the lack of HLA expression. Among the three RCC lines, WT1 antigen was detected in VMRC and TUHR10 cells by RT-PCR and immunohistochemical analysis (Figures 1D and 1E). We then examine the cytotoxic activity of WT1-CTLs by co-culturing them with these target cells. Significant production of IFN-γ by the CTLs was seen only in response to TUHR10 cells, which are HLA-A∗24:02^+^WT1^+^, but not against the other cells (Figure 1F). Furthermore, TUHR10 cells were far more efficiently killed by WT1-CTLs compared with the other cell lines (Figure 1G). These results indicated that WT1-CTLs were able to recognize and kill RCC cells in an antigen-specific manner. It could be pointed out, however, that the WT1-CTLs retain some antigen-non-specific cytotoxic activity, because VMRC and TUHR10 cells were also killed to some extent. Since the WT1-CTLs hardly show cytotoxic activity against K562, which are usually used to assess general NK activity of effector cells, it is probable that VMRC and TUHR10 cells express some molecules inducing NK activity that are not expressed in K562 cells. We thus examined NK cell-associated markers including activating receptors but failed to find difference between WT1-CTLs and peripheral NK cells from healthy donor (Figure 1J).

In order to independently confirm that the cytotoxic activity of the WT1-CTLs is antigen specific, we used a lentivirus system to transduce VMRC cells, which express the WT1 antigen but are negative for HLA-A∗24:02, with an *HLA-A∗24:02* gene, and thus produced VMRC-A∗24 cells expressing a reporter gene (Venus) and HLA-A∗24:02 (Figure 1H). A cytotoxic assay showed that VMRC-A∗24 cells were more efficiently killed than parental VMRC cells by WT1-CTLs (Figure 1I), further confirming that WT1-CTLs kill RCC cells based on recognition of the WT1 antigen.

### Regenerated WT1-CTLs Are Therapeutically Effective in an *In Vivo* Xenograft Model Using a WT1-Expressing RCC Cell Line

**Figure 2:**
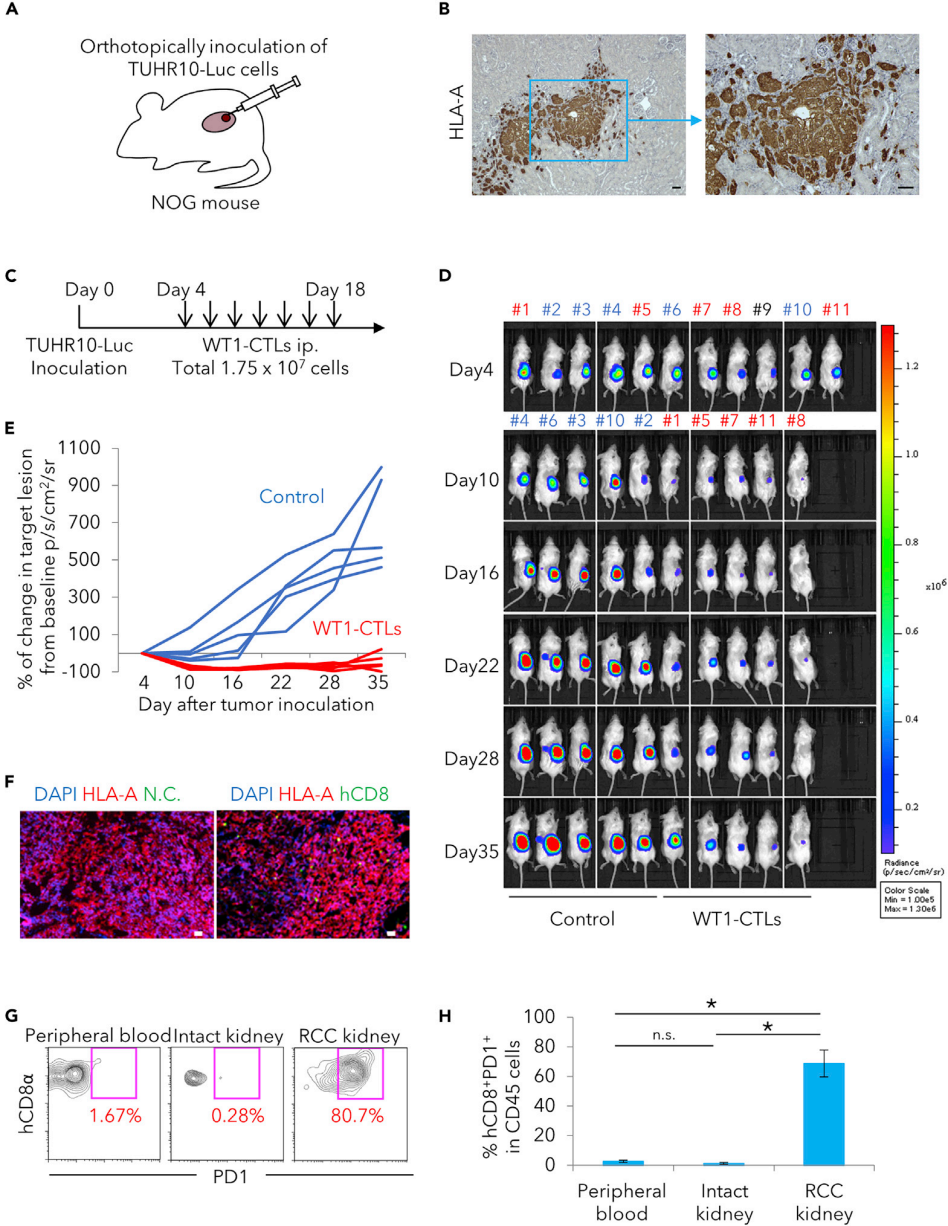
Regenerated WT1-CTLs Have Therapeutic Efficacy in an *In Vivo* Xenograft Model with a WT1-expressing RCC Cell Line (A) Establishment of orthotopic xenograft mouse model by using an RCC cell line expressing WT1 antigen and luciferase, TUHR10-Luc. (B) Immunohistochemistry of a kidney with the tumor lesion. Tumor cells were detected as HLA-A positive. Scale bar, 50 μm. (C) The schedule of the *in vivo* therapeutic experiment. (D) Bioluminescence imaging of control and treatment groups by using an *in vivo* imaging system (IVIS) (n = 5 per group). Cell therapy was begun after the tumors had been allowed to grow for 4 days. Results of one experiment are shown as representative of three independent experiments. (E) Quantification of focal luminescence in the experiment shown in (C) and (D). p/s/cm^2^/sr stands for photons/second/cm^2^/steradian. (F) Immunofluorescence of tumor lesion from mice 2 days after last injection of WT1-CTLs. Tumor cells and CTLs were identified as HLA-A and human CD8^+^ cells, respectively. Left is the image without primary antibody of CD8, and right is with primary antibody of CD8. Right image was used as a negative control (N.C.). Scale bar, 50 μm. (G) Flow cytometry analysis of human CD3, CD45, CD8α, and PD1 expression by peripheral blood lymphocytes, lymphocytes in the intact kidney, and lymphocytes in the RCC-bearing kidney from a treated mouse 2 days after last injection of WT1-CTLs. CTLs from the RCC-bearing kidney had increased expression of PD1. Results of one experiment are shown as representative of three independent experiments. (H) Comparison of the ratio of PD1^+^hCD8^+^ cells normalized by mCD45^+^ cells determined by flow cytometry analysis of peripheral blood cells, lymphocytes in normal intact kidney, and lymphocytes in RCC-bearing kidney from treated mice two days after the last injection of WT1-CTLs (∗p < 0.05). Results are presented as mean ± SD from biological triplicate experiments.

We next investigated whether WT1-CTLs are effective in an *in vivo* xenograft model. TUHR10 cells were first transduced with a *luciferase* gene (hereafter referred to as TUHR10-Luc cells) to make them detectable by a bioluminescence imaging system. TUHR10-Luc cells were orthotopically inoculated inside the kidney of immunodeficient NOG mice (Figure 2A), where they became engrafted and formed a tumor lesion (Figure 2B). In the treatment model, 2.5 × 10^6^ WT1-CTLs plus IL-2, IL-7, and IL-21 were administered intraperitoneally to the tumor-bearing mice a total of seven times from day 4 to day 18 (Figure 2C). Control mice received only cytokines. Reduction of tumor size was clearly seen on day 10, and the tumors remained small until day 28; however, tumor regrowth began to be seen on day 35 (Figures 2D and 2E). Thus, the treatment was highly effective in reducing tumor size and further suppressing tumor growth, although the tumor cells were not completely eliminated.

Some mice in another similarly designed experiment were sacrificed on day 20 (2 days after the last CTL injection), and tumor lesions were histologically examined. Infiltration of CD8T cells was observed in the area, visualized as HLA-A^+^ cells (Figure 2F). Mononuclear cells were harvested from the tumor-bearing and contralateral kidneys and also from peripheral blood and analyzed by flow cytometry. PD1^+^ CD8T cells were detected in the tumor-bearing kidney but not in the control kidney or in peripheral blood (Figures 2G and 2H), suggesting that CTLs in the tumor-bearing kidney had been activated by encountering cognate target cells. CD8T cells detected in the intact kidney were considered to represent circulating CD8T cells, because the profile of these cells was very similar to that of CD8T cells in peripheral blood.

### WT1-TCR-CTLs Regenerated from iPSCs Transduced with TCR α/β Genes

Thus far we have produced CTLs from T-iPSCs that had been originally derived from T cells. Very recently, we have developed a method in which iPSCs originally derived from non-T cells are transduced with exogenous *TCR α/β* genes (hereafter referred to as TCR-iPSCs). This method made it easier to produce iPSCs equivalent to T-iPSCs; hence, hereafter, we used it to produce CTLs.

The use of the TCR-iPSC method enabled us to move our study closer to clinical application, since we could use clinical grade iPSCs and *TCR α/β* genes. We thus selected materials that can be directly applied to clinical settings: (1) iPSCs homozygous for the most frequent Japanese HLA-haplotype (HLA-homo), which had been established for clinical use by the Center for iPS cell Research and Application (CiRA) at Kyoto University, and (2) WT1-specific *TCR α/β* enes (clone name: TAK1) that had already been tested clinically (Tawara et al., 2017). It is expected that CTLs derived from HLA-homo iPS cells encounter minimal immune reaction when they are given to HLA haplotype-heterozygous (HLA-hetero) recipients, since HLA is matched for T cells in recipient in such case.

**Figure 3:**
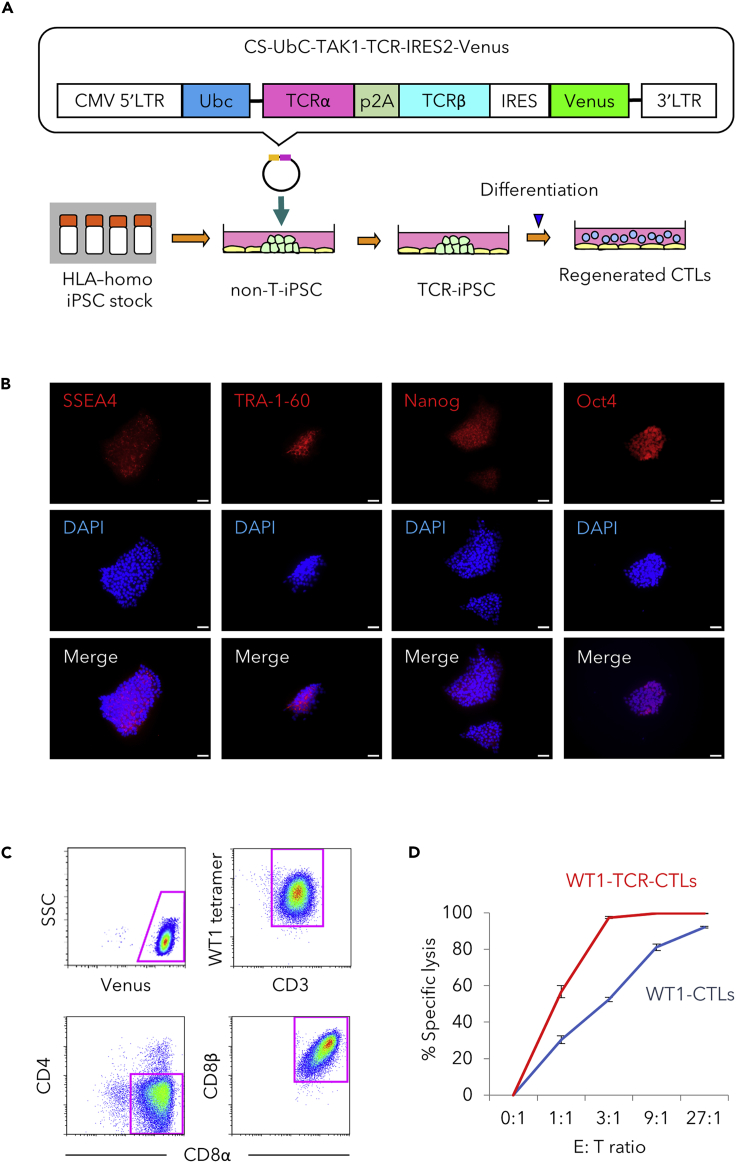
Regenerated WT1-TCR-CTLs from TCR-transduced iPSCs Exhibited Cytotoxic Activity against RCC Cells *In Vitro* (A) Illustrated methods showing the transduction with *TCR α/β* genes specific for WT1 into iPSCs and their differentiation into CTLs. (B) Immunofluorescence analysis for the expression of SSEA4, TRA-1-60, Nanog, and Oct4. DAPI was used as nuclear counter-staining. Scale bar, 50 μm. (C) Flow cytometric profiles of WT1 antigen-specific CD8αβ heterodimer T cells regenerated from HLA-homo iPSCs with WT1-specific *TCR α/β* genes TAK1. Venus was used as a reporter gene. Results of one experiment are shown as representative of three independent experiments. (D) Cytotoxic activity of regenerated CTLs with #3-3 WT1-TCR or TAK1-WT1-TCR against TUHR10-Luc cells at different E:T ratios. Results are presented as mean ± SD from biological triplicate experiments.

HLA-homo iPSCs were transduced with WT1-specific *TCR α/β* genes (TAK1-WT1-TCR) (Figure 3A). WT1-TCR-iPSCs maintained several gene expressions related to pluripotent stem cells (Figure 3B). CTLs regenerated as in Figure 1A were found to almost exclusively express a CD8αβ heterodimer and a WT1-specific TCR (WT1-TCR-CTL) (Figure 3C). The WT1-TCR-CTLs were even more efficient than #3-3 clone WT1-CTLs at killing TUHR10-Luc cells in an *in vitro* cytotoxicity assay (Figure 3D).

### WT1-TCR-CTLs Derived from TCR-iPSCs Have Preclinical Therapeutic Effect against RCC PDX Expressing WT1 Antigen

**Figure 4:**
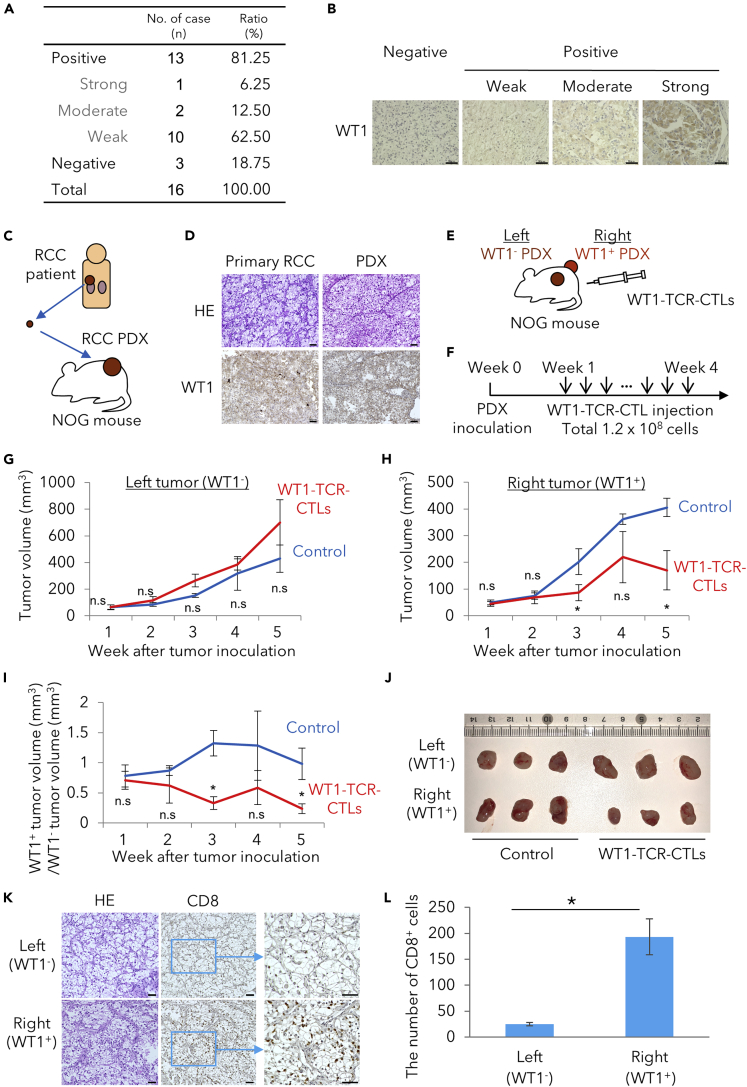
WT1-TCR-CTLs Derived from TCR-iPSCs Have Preclinical Therapeutic Effect Against RCC PDX Expressing WT1 Antigen and HLA-A∗24:02 (A) WT1 antigen expression in clinical samples of clear cell RCC. (B) Immunohistochemical analysis of WT1 expression (negative, weak, moderate, and strong) in clinical samples of clear cell RCC. Scale bar, 50 μm. (C) Illustrated methods showing establishment of subcutaneous RCC-PDX. (D) Comparison of WT1 expression by primary RCC and PDX. Scale bar, 50 μm. (E) The preclinical experimental therapeutic setting. Establishment of RCC-PDX by using WT1^+^ (right) or WT1^−^ (left) tumors from HLA-A∗24:02^+^ patients (n = 3 per group). (F) Schedule of the *in vivo* therapeutic experiment in the PDX model. (G) The WT1^−^ tumor (left) volume in the *in vivo* experiment to examine the therapeutic effect of WT1-TCR-CTLs against RCC-PDX. Results of one experiment are shown as representative of two independent experiments. (H) The WT1^+^ (right) tumor volume in the *in vivo* experiment to examine the therapeutic effect of WT1-TCR-CTLs against RCC-PDX (∗p < 0.05). Results of one experiment are shown as representative of two independent experiments. (I) The WT1^+^ tumor (right) volume normalized by the WT1^−^ tumor (left) volume (∗p < 0.05). Results of one experiment are shown as representative of two independent experiments. (J) An image of resected tumors after treatments. Results of one experiment are shown as representative of two independent experiments. (K) Comparison of infiltrated human CD8T cells in the WT1^−^ tumor (left) and the WT1^+^ tumor (right). Scale bar, 50 μm. (L) Comparison of number of infiltrated human CD8-positive cells per field of view (×20) in the WT1^−^ tumor (left) and the WT1^+^ tumor (right) (∗p < 0.05).

We then histologically analyzed a total of 16 resected RCC specimens from Akita University for WT1 antigen expression and 13 cases were found to be WT1^+^ (Figures 4A and 4B), confirming previous reports that RCC express WT1 antigen (Nakatsuka et al., 2006, Iiyama et al., 2007). For the treatment model, we took the advantage of RCC-PDX, in which tumor tissue resected from a patient with RCC is subcutaneously inoculated into an NOG mouse (Figure 4C). Such RCC-PDX can be maintained by serial transplantation in NOG mice, and several RCC-PDX lines have been established by our group (Inoue et al., 2017). The histological features of the engrafted tumor tissue resemble the original clear cell RCC tissue (Figure 4D) (Pavia-Jimenez et al., 2014). We subcutaneously inoculated an HLA-A∗24:02^+^WT1^+^ PDX tumor and an HLA-A∗24:02^+^WT1^−^ one, the former expressing WT1 antigen at a moderate level and the latter expressing no WT1 antigen as a specificity control, into the right side and left side, respectively, on the back of a NOG mouse (Figure 4E). Regenerated WT1-TCR-CTLs (1 × 10^7^ cells) were then injected intraperitoneally a total of 12 times from week 1 to week 4 (Figure 4F). Suppression of tumor growth, as assessed by tumor volume and visual observation of the surgically resected tumors at the end of the experiment, was seen with the WT1^+^ tumors but not with the WT1^−^ tumors (Figures 4G–4J). Mice were sacrificed on the fifth week, and tumor lesions were histologically examined. Number of infiltrated CD8T cells observed in right WT1^+^ tumor was larger than that in left WT1^−^ tumor (Figures 4K and 4L). These results demonstrated that regenerated CTLs are effective against solid tumors in a PDX model.

## Discussion

In the present study, we have shown that the CTLs produced by using clinical grade iPSCs and clinically tested *TCR α/β* genes are effective against a solid tumor in a PDX model, which is considered to be close to the physiological tumor state (Inoue et al., 2017). The present results thus encourage us to apply our approach to clinical settings.

As the starting cells for the production of CTLs, we utilized two different types of iPSCs, namely, T-iPSCs and TCR-iPSCs, the former being produced by reprogramming antigen-specific T cells and the latter by transducing iPSCs derived from non-T hematopoietic cells with exogenous *TCR α/β* genes. The T-iPSC method can be used both in autologous and allogeneic settings. When the T-iPSC method is used in an allogeneic setting, the original antigen-specific CD8^+^ T cells should be collected from a donor who is HLA-homo, expecting that the CTLs regenerated from the T-iPSCs can be given to a patient heterozygous for the HLA-haplotype (HLA-hetero) (Sugita et al., 2016a, Sugita et al., 2016b). However, such HLA-homo donors are very rare (Okita et al., 2011) and the process to establish a usable T-iPSC clone is expensive and time consuming.

In order to address these issues, we have very recently developed an alternative method, the TCR-iPSC method, where iPSCs are transduced with exogenous *TCR α/β* genes. The merit of this method is that it becomes possible to use iPSCs and *TCR α/β* genes whose quality is guaranteed for clinical use. Hence, in the present study, we decided to use the TCR-iPSC method in our final PDX model experiments, and used an HLA-homo iPSC line provided by CiRA. We also used WT1-specific *TCR α/β* genes (TAK1) that had already been used in a clinical trial (Tawara et al., 2017), rather than the #3-3 WT1-TCR cloned by our group. The regenerated CTLs in this setting were found to exert a strong therapeutic effect in the RCC-PDX model.

Based on the literature and the results presented here, we can estimate the proportion of patients with RCC that could benefit from our TCR-iPSC-derived CTLs. The HLA-homo iPSC line we used can cover ~17% of the Japanese population (Ikeda et al., 2015). Since this haplotype contains HLA-A∗24:02, to which TAK1-WT1-TCR is restricted, this WT1-TCR is always usable when the regenerated CTLs are given to HLA-hetero patients. As shown in the present study, ~80% of RCC specimens expressed the WT1 antigen, but moderately/strongly expressing ones were limited to ~20%. Thus, if regenerated CTLs are applied only to moderately/strongly expressing cases, it can be calculated that 3% of patients with RCC are candidates for the treatment. If we extend our approach by utilizing other HLA-homo iPSC stock lines, covering 33% of Japanese people with the four lines available at present, the proportion of candidate patients would be increased accordingly.

In the present, as well as in our previous studies, we have very carefully investigated whether or not the regenerated CTLs kill target cells in an antigen-specific manner, since it is generally known that CTLs, upon activation, come to express activating receptors associated with NK cells (Themeli et al., 2013, Maeda et al., 2016). Moreover, it was previously shown that CTLs regenerated from iPSCs exhibit a γδT cell-like phenotype (Themeli et al., 2013), endowing them with the potential to kill target cells just like NK cells do. However, in our previous study, we resolved this issue by developing a novel culture method; the CTLs regenerated by the method exhibited only marginal NK cell-like cytotoxicity (Maeda et al., 2016). Even so, we always check whether the observed killing is antigen specific or not. Indeed, the CTLs used in the present study showed virtually no cytotoxic activity against K562 cells, whereas some other HLA-A∗24:02^-^ RCC lines were killed at low frequency (Figure 1G), suggesting that these CTLs retain some NK-like cytotoxicity. Nevertheless, we would argue that RCC cell killing in our PDX model experiment was antigen specific, since the growth of the WT1-negative tumor was not suppressed, whereas that of the WT1-positive tumor in the same mouse was suppressed (Figures 4G–4J).

Recently, another group has reported that they regenerated CTLs expressing the same TAK1-WT1-TCR as we used in the present study, by using a similar TCR-iPSC method. The regenerated CTLs were shown to be effective in suppressing the growth of NCI-H266 (human lung adenocarcinoma cell line) cells in a xenograft model (Minagawa et al., 2018). However, the studies did not assess whether suppression of tumor growth was antigen specific or not. Moreover, a cancer cell line was used while we used the more physiological PDX model. Thus, the advantages of the present study are (1) antigen-specific cytotoxicity was demonstrated and (2) a PDX model instead of cell line was used.

In summary, in the present study, we demonstrated that the CTLs regenerated by the T-iPSC method effectively inhibited the growth of an RCC cell line in a xenograft model and CTLs regenerated by the TCR-iPSC method utilizing clinically applicable materials were capable of suppressing the growth of RCC tumors in a patient-derived xenograft model. We propose that this method to produce CTLs from pluripotent stem cells is applicable against solid tumors.

### Limitations of the Study

There are some limitations of our method that are worth discussing here. In our treatment model, tumors were not completely eliminated but survived in both the RCC cell line and PDX models. Since this kind of limitation is generally seen in any immunotherapies that target a specific tumor antigen, resolution of this issue is beyond the scope of the present study. In this context, we are now developing adjunct therapy that can enhance the anti-tumor effect in our PDX treatment model, such as blocking immune checkpoint signals. In addition, it is important to think of tumor microenvironment, which is generally immuno-suppressive, mainly by regulatory T cells. In the present study, however, we transferred only CD8^+^ CTLs into xenograft model and we used NOG mice as recipients, which do not have any T cells. Therefore, it is possible that we will face this issue when our strategy goes into clinical application.

## Methods

All methods can be found in the accompanying Transparent Methods supplemental file.

## Supplemental Information

Document S1. Transparent Methods
