# Supplementary material for: Cytotoxic T Lymphocytes Regenerated from iPS Cells Have Therapeutic Efficacy in a Patient-Derived Xenograft Solid Tumor Model
Source: iScience. 2020 Apr 6;23(4):100998. doi: 10.1016/j.isci.2020.100998 (PMC7188741; doi:10.1016/j.isci.2020.100998)
Supplement: Document S1. Transparent Methods [file mmc1.pdf]

**iScience, Volume *23***

## Supplemental Information

# Cytotoxic T Lymphocytes Regenerated

# from iPS Cells Have Therapeutic Efficacy

# in a Patient-Derived Xenograft Solid Tumor Model

**Soki Kashima, Takuya Maeda, Kyoko Masuda, Seiji Nagano, Takamitsu Inoue, Masashi Takeda, Yuka Kono, Takashi Kobayashi, Shigeyoshi Saito, Takahiro Higuchi, Hiroshi Ichise, Yuka Kobayashi, Keiko Iwaisako, Koji Terada, Yasutoshi Agata, Kazuyuki Numakura, Mitsuru Saito, Shintaro Narita, Masaki Yasukawa, Osamu Ogawa, Tomonori Habuchi, and Hiroshi Kawamoto**

## Transparent Methods

### Study approval

This study was approved by the institutional review board of the Graduate School of Medicine, Kyoto University (approval number: G52, G761, G793), Akita University (approval number: 1479) and abided by the tenets of the Declaration of Helsinki. All specimens from healthy individuals and patients were collected after written informed consent was obtained.

### Cell lines

OP9, OP9/DLL1, A498, VMRC-RCW (VMRC), and TUHR10-TKB (TUHR10) were purchased from RIKEN BRC. LCLs were established from the same healthy volunteer from which the T-iPSCs were derived. K562 was a gift from Dr. Kiyotaka Kuzushima (Aichi Cancer Center). The cell lines were authenticated by morphology, growth rate and surface phenotype, and especially expression of HLA class I, except for K562, which is HLA-negative.

### Flow cytometry

Single cell suspensions from the regenerated CTLs, cell lines, mouse peripheral blood, spleen and kidney were stained with the following: PE-Cy7- or APC- or V450-hCD3e (UCHT1), FITC- or APC- or APC-Cy7- or V450-hCD8α (HIT8α, RPA-T8), BV421-hCD56 (HCD56), APC-DNAM1 (11A8), PE-NKG2D (1D11), PE-NKp30 (P30-15) and BV421-hPD1 (EH12.2H7) were purchased from BioLegend. APC-hCD8β, FITC-mCD45 were purchased from BD Bioscience. PE-Cy7-hCD4 (RPA-T4) was purchased

from TONBO. PE-HLA-A*24:02 modified WT1 tetramer and anti-HLA-A24 mAbs (17A10) were obtained from MBL. Alexa Fluor488-NKG2C (134591), APC-KIR2DL1 (143211) and PE-KIR2DL3 (180701) were purchased from R&D. Data was acquired by FACSCanto™ and were analyzed with FlowJo software (BD Biosciences).

**Isolation of hematopoietic cells from kidney**

Systemic mouse blood was refluxed with 50ml PBS prior to collecting infiltrating hematopoietic cells in the kidney. The kidney was then harvested and dissociated using scissors and gentleMACS™ Dissociators (Miltenyi Biotec). 30% Percoll gradient centrifugation was used to exclude cells except hematopoietic cells (GE Healthcare Life Sciences).

**Establishment of exogenous gene expressing cell lines**

VMRC-A*24 cells were established by lentiviral transduction. In brief, HLA-A*24:02:01 cDNA clones were provided by the RIKEN BRC through the National Bio-Resource Project of the MEXT, Japan and cloned by methods described previously (Akatsuka et al., 2002). We prepared lentivirus vector CS-UbC-RfA-IRES2-Venus (a gift from Hiroyuki Miyoshi, Keio University and Atsushi Miyawaki, RIKEN) including the HLA-A*24:02:01 (Ichise et al., 2017). cDNA clone of *HLA- A*24:02:01* was subcloned into the pENTR/D-TOPO vector and further subcloned into an expression vector, CS-UbC-RfA-IRES2-Venus using pENTR Directional TOPO Cloning Kits (Thermo Fisher Scientific). Lentiviruses were collected 48 hr after transfection of Lenti-X 293T cells (Clontech) with appropriate amounts of lentiviral vectors, pRSV-Rev, pMDLg/pRRE, and pMD2.G (Addgene) using ViaFect (Promega). $5\times10^4$ VMRC cells were transduced and

Venus-expressing cells were sorted by FACSAriaII™ (BD Bioscience) and seeded onto culture dishes. TUHR10-Luc cells were established by lentiviral transduction of pHIV-Luc-ZsGreen (Plasmid #39196, Addgene) and ZsGreen-expressing cells were sorted by FACSAriaII™ (BD Bioscience) and seeded onto culture dishes.

**RNA Extraction and cDNA synthesis by reverse transcription**

Total RNA from cell lines was isolated using the RNeasy Mini Kit following the manufacturer's instructions (Qiagen). The concentration and 260:280 nm ratio of the extracted RNA was determined using spectrophotometry NanoDrop (Thermo Fisher Scientific). cDNA synthesis was performed using a SuperScript VILO cDNA Synthesis Kit (Thermo Fisher Scientific) from 2.5 μg of total RNA template following the manufacturer's instructions.

**RT-PCR**

The PCR reactions were performed using KOD -Plus- ver.2 (TOYOBO) as follows: 10 min at 95 °C followed by 35 cycles consisting of 15 s at 95 °C, 60 s at 63 °C and 30 s at 68 °C, and finally 10 min at 68°C. Amplified products were analyzed by agarose gel electrophoresis. The primers and probes were synthesized by Sigma-Aldrich. *WT1*: forward; 5’-ACAGGGTACGAGAGCGATAACCA-3’, reverse; 5’-CACACGTCGCACATCCTGAAT-3’ (Willasch et al., 2009). *GAPDH*: forward; 5’-GAAGGTGAAGGTCGGAGTC-3’, reverse; 5’-GAAGATGGTGATGGGATTTC-3’.

**Immunohistochemical staining**

For figure 1E, each cell line was inoculated subcutaneously into a NOD/Shi-scid,IL-

2RγKO Jic (NOG) mouse on day 0. When the tumor size reached around 1cm in diameter, tumors were resected, formalin-fixed and paraffin-embedded (FFPE). For figure 2B, TUHR10-Luc was orthotopically inoculated into the NOG mouse kidney. On day 7, the tumor-bearing kidney was resected. For figure 4B, FFPE tissues from 16 clear cell RCC patients were retrieved from archive sources at Akita University Hospital. For figure 4D, FFPE tissues from primary clear cell RCC or from PDX were retrieved from archive sources at Kyoto University Hospital. Immunohistochemistry was performed on 4-μm-thick FFPE sections as previously described in (Nakatsuka et al., 2006) with slight modifications. Briefly, sections were treated with a 0.3% $H_2O_2$ solution to reduce endogenous peroxidase activity. After incubation with 10% proteinase K at RT, sections were subjected to heat-induced epitope retrieval in Target Retrieval Solution (Code No. S1699; Dako), followed by incubation with anti-WT1 antibody (clone 6F-H2; Dako) diluted as 1:100, negative control mouse IgG1 (Code No. X0931; Dako) diluted as 1:130, anti-HLA-A antibody (clone EP1395Y; abcam) diluted as 1:200 or negative control rabbit IgG (clone EPR25A; abcam) diluted as 1:100 overnight at 4°C. WT1 staining visualization was performed by using HRP-DAB (Code No. K5007; Dako) and counterstaining with hematoxylin. DAB staining was stopped when a positive signal was detected in podocytes of mouse kidney as a positive control. Hematoxylin and eosin staining was performed by the Kyoto Institute of Nutrition & Pathology or the Center for Anatomical Studies in Kyoto University Graduate School of Medicine. Images were obtained using a Keyence BZ-9000. WT1 positive area was calculated using BZ-II Analyzer Ver. 1.42 (KEYENCE).

**Immunofluorescence staining**

For Figure 2F, tumor inoculated kidney from mice were embedded in Tissue-Tek OCT compound (SAKURA FINETEK), and snap frozen in liquid nitrogen. 4-µm-thick cryostat sections were prepared and placed on APS-coated glass slides. Sections were fixed with acetone (nacalai tesque) for 4 min at 4°C, dried, and kept at −80°C until use. For Figure 3B, iPS cells colonies were seeded on chamber slides (IWAKI), and then fixed with acetone (nacalai tesque) for 4 min at 4°C and dried. After blocking with Blocking One Histo (nacalai tesque) for 30 min at RT, sections were incubated for 1 h at RT with anti-hCD8 (Dako), anti-HLA-A (Abcam), anti-SSEA4 (Abcam), anti-TRA-1-60 (Abcam), anti-Nanog (Abcam), or anti-Oct4 (Abcam) mAbs or isotype control diluted as 1:100 (hCD8, HLA-A and Oct4), 1:200 (SSEA4 and TRA-1-60) and 1:1000 (Nanog) by 0.5 % tween 20 PBS with blocking buffer, and washed with PBS five times. Sections were incubated for 30 min at RT with DAPI diluted as 1:1000 and Alexa fluor 546 or Alexa fluor 488 diluted as 1:200 or 1:100 by 0.5 % tween 20/PBS with blocking buffer, and washed with PBS five times. In figure 4L, the number of CD8-positive cells was counted in a field of view (x20) at three different locations in the $WT1^-$ tumor (left) and the $WT1^+$ tumor (right). Images were obtained using a Keyence BZ-9000.

**Construction of WT1-TCR lentivirus vector and transduction to HLA haplotype-homozygous iPSCs**

WT1-specific *TCR α* and *β* genes of TAK1 clone were obtained from Dr. Yasukawa in Ehime University. *TCR α* and *β* cDNAs were linked with the self-cleaving P2A sequence and subcloned into pENTR/D-TOPO vector and further subcloned into an expression vector, CS-UbC-RfA-IRES2-Venus (gifted from Hiroyuki Miyoshi, Keio University and Atsushi Miyawaki, RIKEN) using pENTR Directional TOPO Cloning Kits (Thermo

Fisher Scientific). Culture supernatant containing lentiviruses were collected 48–72 h after transfection of Lenti-X 293T cells (Clontech) with appropriate amounts of lentiviral vectors, pRSV-Rev, pMDLg/pRRE and pMD2.G (Addgene) using ViaFect (Promega). The HLA homozygous iPSC (clone name: FFI14s04) was obtained from CiRA, Kyoto University. $5\times10^4$ iPSCs were transduced with lentivirus by centrifugation (32 °C, 600G, 1h) and seeded on a 6 well-plate with StemFit AK02 medium (Ajinomoto). TAK1-TCR-transduced iPSCs as venus-positive cells were single-cell-sorted by FACSMelody™ (BD Bioscience) into 96 well-plate and established as independent lines.

**Regeneration of CD8$^+$ T cells from iPSCs *in vitro***

CD8 single-positive T cells were regenerated from iPSCs using the OP9 and OP9/DLL1 stromal cell co-culture systems (Vizcardo et al., 2013; Maeda et al., 2016). About 600 human iPSC clumps were plated on gelatin pre-coated OP9 overconfluent 10 cm dishes filled with 10 ml of OP9 medium, i.e. α-MEM (Invitrogen) with 20% FCS, penicillin (100 U/mL), and streptomycin (100 μg/mL). On the next day, medium was replaced by 20 ml of fresh medium and thereafter changed every 4 days. On day 13, colonies were treated for 45 minutes with 10 ml of collagenase Type IV (50 U/ml) (Invitrogen) and subsequently dissociated for 30 minutes at 37°C using trypsin-EDTA (0.05%) (Nacalai tesque). To remove stromal cells, dissociated cells were resuspended by adding 5 times v/v OP9 medium and then plated on plastic at 37°C for one hour and floating cells were collected. To remove any remaining stromal cells and aggregated cells, the cell suspensions were passed through a 100 μm filter. Cells were plated in an OP9/DLL1 semi-confluent dish in OP9 medium containing hIL-7 (5 ng/ml), hFlt-3L (5 ng/ml), and hSCF (5 ng/ml). On day 16, semi-adherent cells were collected and passaged into a new dish

layered with OP9/DLL1 cells. From this point, passage was done every 7 days. On day 35, floating cells were collected. CD4/8 DP cells were enriched by using CD4 microbeads (Miltenyi Biotec). DP cells were stimulated with 50 ng/ml CD3 antibody (OKT-3) (eBioscience) in the presence of hIL-2 (100 U/ml) and hIL-7 (5 ng/ml). Regenerated CTLs were expanded by co-culture with modified WT1 peptide (CYTWNQMNL, Eurofins Genomics) (Tsuboi et al., 2002) loaded LCL in the presence of and hIL-7 (5 ng/ml), hL-21 (10ng/ml) and vitamin C (100 uM) once a week.

**_In vitro_ cytokine release assay using ELISA**

The regenerated CTLs were tested for reactivity in IFNγ release assays with a Human IFN gamma ELISA Ready-SET-Go!™ Kit (Code No. 88-7316; Affymetrix), following the manufacturer's instructions. Effector cells and target cells ($1 \times 10^5$ cells for each) were co-cultured for 5h in ninety-six-well V-bottomed plates with technical-triplicate wells. Cytokine secretion was measured in culture supernatants diluted to fall within the linear range of the assay. Absorbance at 450 nm was measured using a SpectraMax i3 (Molecular Devices).

**_In vitro_ cytotoxicity assay using a $^{51}$Cr release assay**

Regenerated CTLs were used as effector cells in $^{51}$Cr release assays against target cells, LCL or RCC cell lines. LCLs were pulsed with WT1 peptide at various concentrations. $^{51}$Cr-labeled target cell number was fixed 5,000 cells. Target cells and effector cells were co-cultured in 96 well V-bottomed plates (Nunc) at various E:T ratios as indicated. After co-culture, culture supernatant was applied to Picoplates (PerkinElmer) and analyzed by TopCount NXT (PerkinElmer). The percentage of specific lysis was calculated as follows:

Specific lysis (%) = (sample lysis with CTLs (%) − basal lysis without CTLs (%)) / (100 − basal lysis without CTLs (%)).

***In vitro* cytotoxicity assay using a luciferase assay**

The comparison of cytotoxic activity by regenerated CTLs with #3-3 TCR or TAK1-TCR was determined by a standard luciferase-based assay (Eyquem et al., 2017). TUHR10-Luc were used as target cells at different E:T ratio. The effector and target cells were co-cultured in triplicates at the indicated E: T ratio using black-walled 96- well plates with $1 \times 10^5$ target cells in a total volume of 100 μl per well. Target cells alone were plated to determine the maximal luciferase expression (relative light units; RLUmax). 16 h later, luciferase substrate (Bright-Glo™ Luciferase Assay System, Promega) was directly added to each well. Emitted light was detected in a luminescence plate reader GloMax™ (Promega). Lysis was determined as (1 − (RLUsample)/(RLUmax)) × 100.

***In vivo* treatment model using a cell line-derived xenograft mouse**

NOG female mice were purchased from In-Vivo Science Inc. All mice were 6 to 10 weeks old at the beginning of each experiment. Animal studies were performed in compliance with the Institutional Animal Care and Use Committee regulations of Kyoto University (approval number: K-16-11-5). Orthotopic transplantation with RCC cell lines was performed as previously reported (Karashima et al., 2017). On day 0, $4 \times 10^6$ TUHR10-Luc cells were injected in the right kidney of mice. #9 mouse was excluded before the treatment, because the tumor of the mouse was very low intensity of radiance. On day 4, tumor engraftment was confirmed using an *in vivo* imaging system (IVIS) with Living Image software (PerkinElmer). Then each mouse uniformly allocated to control group or

CTL treatment group based on the radiance score of the region of interest. After allocation, the mice in the treatment group were intraperitoneally injected with 2.5 × $10^6$ WT1-CTLs and cytokines (IL-2 160U, IL-7 40ng and IL-21 40ng /body), and the mice in control group were intraperitoneally injected with cytokines three times a week for a total of seven times. We used the method of intraperitoneal (i.p.) injection to mice based on the previous report showing that intravenous injection is comparable i.p. injection in cancer immunotherapy model (Petersen et al., 2006; Friedrich et al., 2012; Minagawa et al., 2018). The radiance scores of tumors were measured every week on the IVIS. Two days after the last treatment day, tumor infiltrating cells in the right kidney, left normal kidney infiltrating cells and peripheral blood were analyzed by flow cytometry.

***In vivo* treatment model using the RCC-PDX mouse**

RCC tumor samples were obtained from patients after obtaining informed consent for sample procurement as approved by Kyoto University Hospital. Eligibility criteria were based on preoperative computed tomography (CT) scan and included tumor samples greater than 1 cm. A tumor sample was subcutaneously inoculated into mice. Tumor size was measured by a digital caliper once per week. When the tumor size reached around 1 cm diameter, mice were anesthetized with isoflurane and sacrificed for ethical considerations. When tumor passage was needed, fragmented tumors were transplanted into mice. On day 0, 5 × 5 × 5 mm tumor fragments were inoculated into 6-14 weeks old NOG female mice. A WT1-positive tumor was inoculated in the right flank and a WT1-negative tumor was inoculated in the left flank. On day 7, tumor engraftment was confirmed and then each moue was allocated to the control group or WT1-TAK1-CTL treatment group. After allocation, the mice in the treatment group were intraperitoneally

injected with 1 × $10^7$ CTLs and cytokines (IL-2 160U, IL-7 40ng and IL-21 40ng /body) and the mice in the control group were intraperitoneally injected with the cytokines every other day for a total of 12 times. The tumor size was measured by a digital caliper once per week. On the seventh day after the last treatment day, mice were euthanized and tumors were resected.

**Statistical analysis**

All statistical analyses were performed using Statcel (OMS Ltd., Tokyo, Japan). Comparisons between groups were performed with student't test or $\chi^2$ test, as appropriate.

## Supplemental References


Akatsuka, Y., Goldberg, T.A., Kondo, E., Martin, E.G., Obata, Y., Morishima, Y., Takahashi, T. and Hansen, J.A. (2002). Efficient cloning and expression of HLA class I cDNA in human B-lymphoblastoid cell lines. Tissue Antigens *59*, 502-511.

Eyquem, J., Mansilla-Soto, J., Giavridis, T., van der Stegen, S.J., Hamieh, M., Cunanan, K.M., Odak, A., Gonen, M. and Sadelain, M. (2017). Targeting a CAR to the TRAC locus with CRISPR/Cas9 enhances tumour rejection. Nature *543*, 113-117.

Friedrich, M., Raum, T., Lutterbuese, R., Voelkel, M., Deegen, P., Rau, D., Kischel, R., Hoffmann, P., Brandl, C., Schuhmacher, J. (2012). Regression of Human Prostate Cancer Xenografts in Mice by AMG 212/BAY2010112, a Novel PSMA/CD3-Bispecific BiTE

Antibody Cross-Reactive With Non-Human Primate Antigens. Mol Cancer Ther *11*, 2664-2673.

Ichise, H., Nagano, S., Maeda, T., Miyazaki, M., Miyazaki, Y., Kojima, H., Yawata, N., Yawata, M., Tanaka, H., Saji, H., et al. (2017). NK Cell Alloreactivity against KIR-Ligand-Mismatched HLA-Haploidentical Tissue Derived from HLA Haplotype-Homozygous iPSCs. Stem Cell Reports *9*, 853-867.

Karashima, T., Udaka, K., Niimura, M., Suzuki, K., Osakabe, H., Shimamoto, T., Fukata, S., Inoue, K., Kuroda, N., Seiki, M., et al. (2017). Therapy with transcutaneous administration of imiquimod combined with oral administration of sorafenib suppresses renal cell carcinoma growing in an orthotopic mouse model. Oncol Lett *14*, 1162-1166.

Petersen, C.C., Petersen, M.S., Agger, R., Hokland, M.E. (2006). Accumulation in Tumor Tissue of Adoptively Transferred T Cells: A Comparison Between Intravenous and Intraperitoneal Injection. J Immunother *29*, 241-249.

Tsuboi, A., Oka, Y., Udaka, K., Murakami, M., Masuda, T., Nakano, A., Nakajima, H., Yasukawa, M., Hiraki, A., Oji, Y., et al. (2002). Enhanced induction of human WT1-specific cytotoxic T lymphocytes with a 9-mer WT1 peptide modified at HLA-A*2402-binding residues. Cancer Immunol Immunother *51*, 614-620.

Willasch, A.M., Gruhn, B., Coliva, T., Kalinova, M., Schneider, G., Kreyenberg, H., Steinbach, D., Weber, G., Hollink, I.H., Zwaan, C.M., et al. (2009). Standardization of WT1 mRNA quantitation for minimal residual disease monitoring in childhood AML and

implications of WT1 gene mutations: a European multicenter study. Leukemia *23*, 1472-1479.
